# Weir’s Index to the Medical Press

**Published:** 1896-04

**Authors:** 


Weir's Index to the Medical Press. Published monthly. Subscription price, $3.00 a year; single copy, 25 cents.

This is a new journal which will be a complete index to all the medical literature published every month. It will be of invaluable service to the medical profession in keeping up with the literature of the day.

The initial, and each successive issue, will treat the entire medical literature of the month immediately preceding as one vast volume, to which it will aim to be the Index or Contents Table. For this purpose, an editorial staff—the personnel of which has been carefully chosen, in order to assure prompt and accurate work—will review monthly the entire medical press of the United States and Canada, including in addition to the published transactions of the various National and State Medical Societies, the current number of every important medical periodical published in the two countries. The result of its labors will be presented in the form of a monthly magazine of from 112 to 128 pages, to be known as *Weir's Index to the Medical Press.*

